# Eclecticism Defined

**Published:** 1872-11

**Authors:** 


### Eclecticism Defined.

The Iowa State Eclectic Medical Association recently passed the following resolutions :

WHEREAS, The term Eclecticism has been variously construed by different parties; and, whereas, the clear enunciation of our principles from time to time will enable the medical fraternity, and the people, to better understand our system of practice; therefore,

*Resolved*, As the sense of this convention, that the total rejection of venesection, arsenic, and antimony, and all their compounds and preparations, as well as lead and copper, and all their compounds and preparations, except as topical applications, are now and ever have been radical and cardinal features of our system of practice.

*Resolved*, That we doubt the propriety of introducing into the diseased human body, metals which compose no part of it in health, and which are slowly and imperfectly eliminated, and insist on the superiority of organic remedies and those metals found in the normal human body.

We cannot find in these resolutions a "peg on which to hang one's hat." Certainly there are no principles enunciated upon which to base a system of medicine. Very many regular physicians never order venesection or prescribe arsenic, corrossive sublimate, etc., and employ antimony very sparingly, if at all. In fact we know regular physicians of large practice, who, probably, do not make use of that great bug-bear among eclectics, calomel, half a dozen times in a year. "Allopathists" differ more in their views and modes of practice among themselves, than is set forth in these resolutions as the difference between eclecticism and regular medicine.

But the "minerals" which the eclectics *taboo* per *orem* they make use of topically. But what is the difference in the action of a medicine on the system, whether it is introduced through the stomach or through the skin or any free surface? Arsenic will as certainly kill when introduced topically in proper doses as when given by the mouth.

It is stated that the propriety is doubted of introducing into the diseased human body, metals which compose no part of it in health. But why not as well introduce metals into the body which are not found naturally there, as to introduce other materials that are not found in the healthy body? What more objections can there be in employing antimony internally, for certain effect, than there are in making use of alcohol to bring about particular results. The latter no more composes a part of the body than the former. And so with many other remedies that eclectics employ. But if a medicine can be made use of properly externally upon the skin, or an ulcerated surface, why not internally upon the mucous membrane of the stomach or intestines? When lead is exhibited by a regular physician it is generally in small doses, in dysentery, etc., for its astringent effect—in other words, as an *internal* topical agent. And why not, if it can be employed externally? Supposing, even, it has to be absorbed and conveyed by the blood in order to reach a part where its action is needed? What harm is done? The blood is all the time, in health, acting as a sort of a sewer in conveying along its channel effete materials and combinations formed in the body, that do not compose a part of the body. This it does, too, without detriment—it is a part of its office.

Eclecticism may succeed very well among the ignorant who are unable to detect a very transparent folly, but it can have no success among intelligent people. The latter are not scared by the cry of "mineral poison," knowing, as they do, that the most virulent and the most to be dreaded, are furnished by the vegetable world.—*Cincinnati Medical News.*

